# Colonic Obstruction from an Unusual Cause: A Rare Case of Metastatic Invasive Ductal Carcinoma to the Colon

**DOI:** 10.7759/cureus.2588

**Published:** 2018-05-07

**Authors:** Heather Katz, Hassaan Jafri, Rahoma Saad, Teresa Limjoco, Maria T Tirona

**Affiliations:** 1 Hematology/oncology, Marshall University, Joan C. Edwards School of Medicine; 2 Internal Medicine, Marshall University Joan C. Edwards School of Medicine; 3 Internal Medicine, Marshall University, Joan C. Edwards School of Medicine; 4 Pathology, Cabell Huntington Hospital and Marshall University, Huntington, West Virginia, Usa; 5 Director of Medical Oncology, Marshall University, Joan C. Edwards School of Medicine

**Keywords:** metastatic invasive ductal carcinoma, colon metastasis, colonic obstruction

## Abstract

Colon metastasis from breast cancer is rare. Gastrointestinal (GI) metastasis is more frequently seen in patients with invasive lobular carcinoma of the breast compared to invasive ductal carcinoma; however, the most common sites of metastasis still remain the lymph nodes, lungs, liver, and bones. We describe a 68-year-old female with a remote history of invasive ductal carcinoma of the breast who presented with abdominal pain and a palpable mass. On imaging, she was found to have a colonic obstruction and underwent a right hemicolectomy that proved to be metastatic invasive ductal carcinoma of the breast.

## Introduction

Breast cancer is the most commonly diagnosed cancer in women in the United States and is the second most common cause of death in women after lung cancer [1-6]. Metastatic breast cancer, although incurable, can be treated with the intention to prolong survival. The most common locations for metastasis from breast cancer include the lymph nodes, lungs, liver, and bone [3-4, 6].

Metastatic breast cancer to the colon is rare [1-4, 6-7]. Metastasis to the gastrointestinal (GI) tract presents variably, ranging from abdominal pain to obstruction. Most reported cases of metastatic disease to the colon are from invasive lobular carcinoma [2-6, 8]. Few case reports have described metastasis from invasive ductal carcinoma.

In this case report, we describe a patient with a history of invasive ductal carcinoma that had metastasized only to bone who presented 15 years later with a colonic obstruction due to a mass. It behaved like a primary colon cancer but pathologically was determined to be metastatic invasive ductal carcinoma of the breast.

## Case presentation

A 68-year-old female presented with right upper quadrant abdominal pain and swelling. The pain was described as sharp, intermittent pain with 10/10 severity that started the day prior to admission. It was not associated with food, and she denied any fevers or chills. Her last bowel movement was the day prior to admission and was of normal caliber. She denied diarrhea, melena, hematochezia, and unintentional weight loss.

She had a past medical history of estrogen receptor (ER), progesterone receptor (PR), and human epidermal growth factor 2 (HER2)-positive invasive ductal carcinoma of the left breast diagnosed in 1996. She received a modified left radical mastectomy and right simple mastectomy, adjuvant systemic chemotherapy with doxorubicin and cyclophosphamide, radiation to the left chest wall, and was started on Tamoxifen. Five years later, she developed metastatic disease to the lumbar spine (ER+/PR+) and received palliative radiation. She was started on zoledronic acid for bone metastasis and her endocrine therapy was switched to letrozole. At the time of admission, she was currently on letrozole and methadone for her back pain. She denied any tobacco use, alcohol, or recreational drug use. She has no family history of cancer.

**Figure 1:**
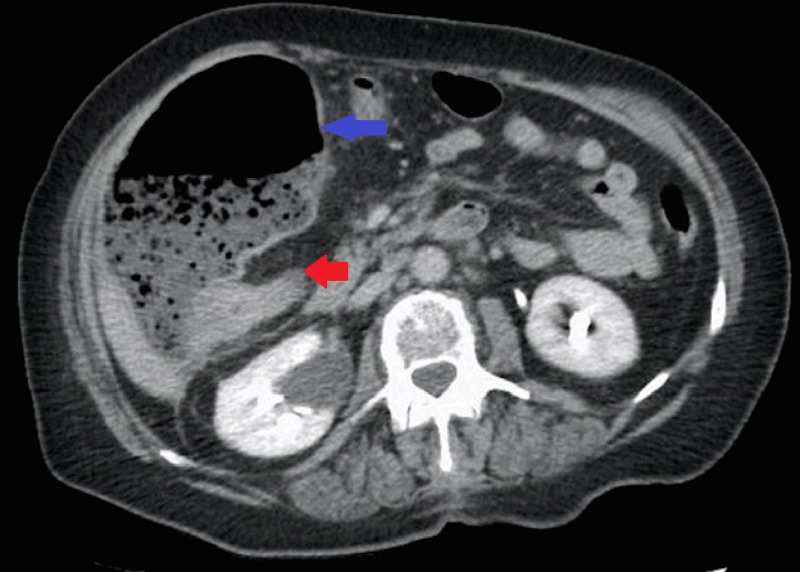
CT abdomen/pelvis Computed tomography (CT) of the abdomen and pelvis demonstrating a colonic mass (red arrow) with dilated colon (blue arrow) indicating obstruction.

Physical exam revealed stable vital signs and tenderness in the right upper quadrant with a large mass palpated. Laboratory data were unremarkable. Computed tomography of the abdomen and pelvis showed a colonic mass measuring 3.5 centimeters in the hepatic flexure, causing obstruction of ascending colon (Figure 1).

A colonoscopy was performed and showed mild diverticulosis in the colon along with petechiae in the proximal to mid-transverse colon likely representing brief ischemia from possible volvulus, but no obvious mass was appreciated. Surgery was consulted due to worsening pain and abdominal swelling and a right hemicolectomy with ileocolic anastomosis was performed.

**Figure 2:**
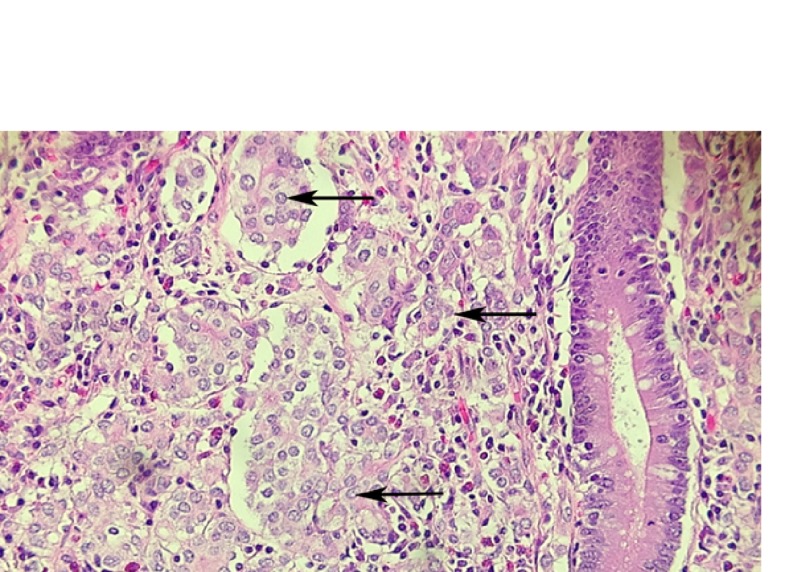
Hematoxylin & eosin (H&E) stain Metastatic tumor cell clusters (arrows) within colon lamina propria; adjacent colonic crypt.

Pathology revealed metastatic carcinoma measuring 6.5 centimeters in greatest dimension at the junction of the cecum and ascending colon and involved the appendix (Figure 2).

**Figure 3:**
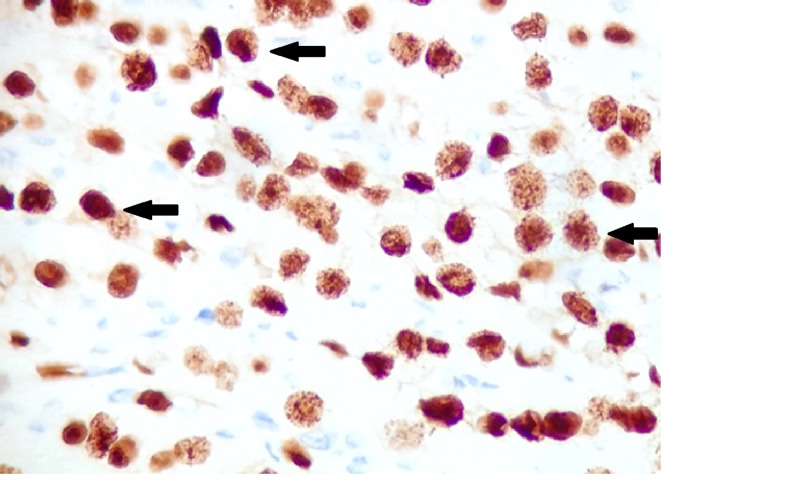
Immunohistochemistry for estrogen receptor Positive nuclear staining for estrogen receptor (arrows indicate examples of positive stained cells).

**Figure 4:**
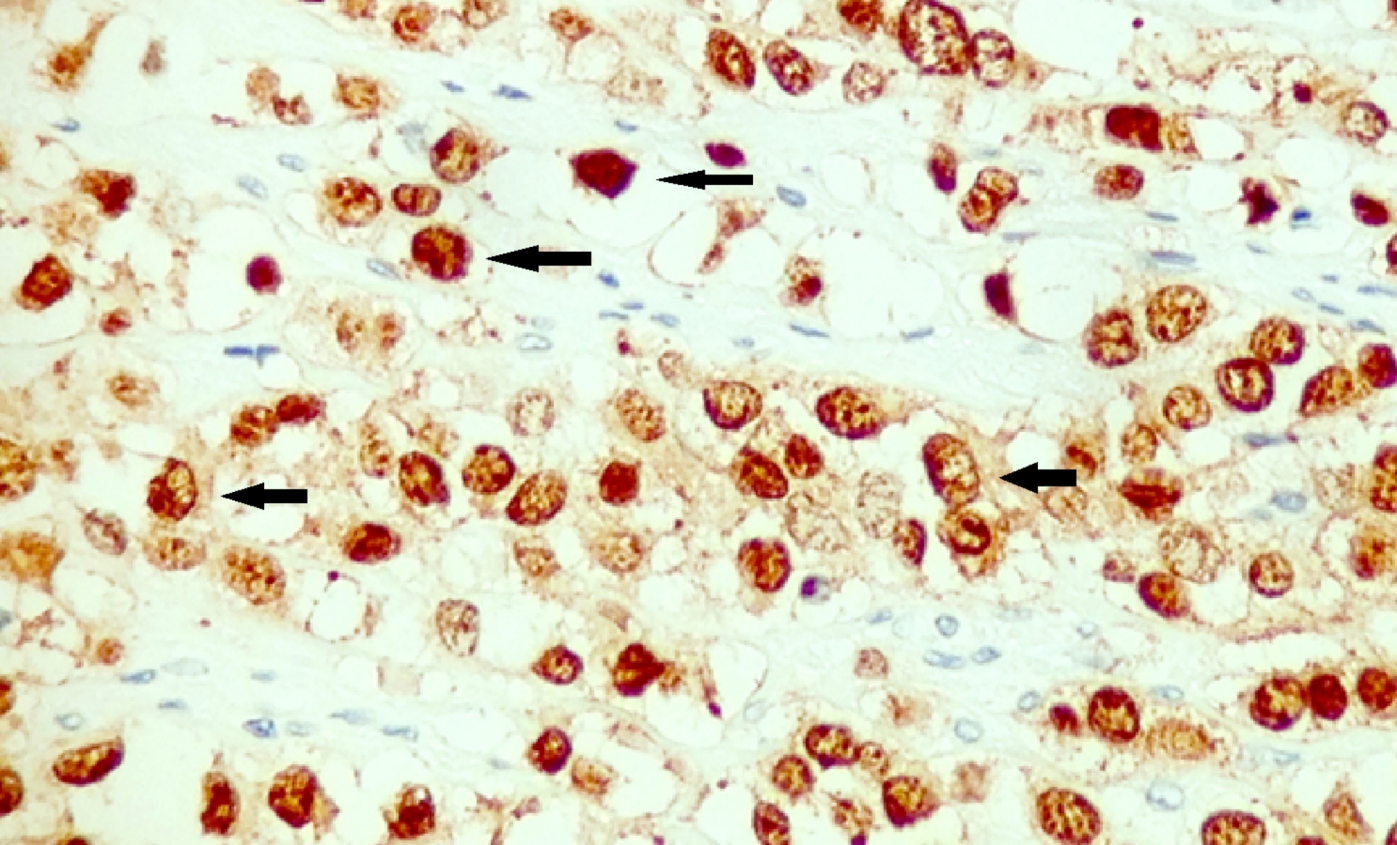
Immunohistochemistry for GATA-3 Positive nuclear staining for GATA-3 (arrows indicate examples of positive staining).

**Figure 5:**
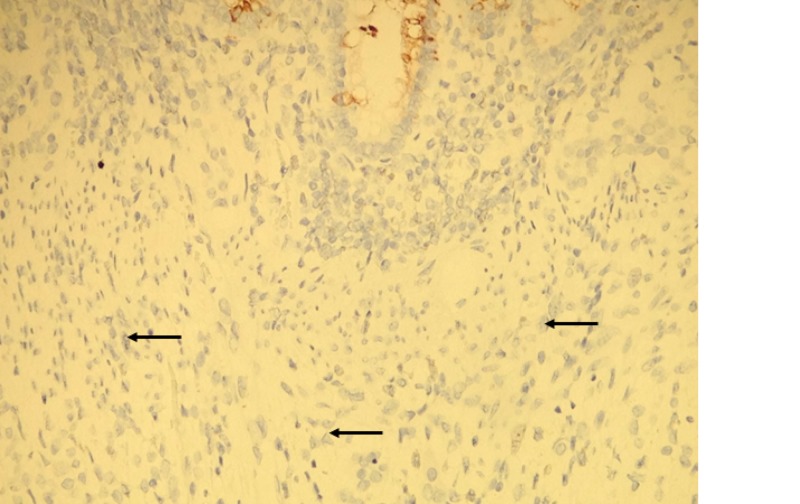
Immunohistochemistry for cytokeratin 20 Tumor cells are negative with cytokeritin 20 (arrows). Colonic crypt cells are positive (top of image).

**Figure 6:**
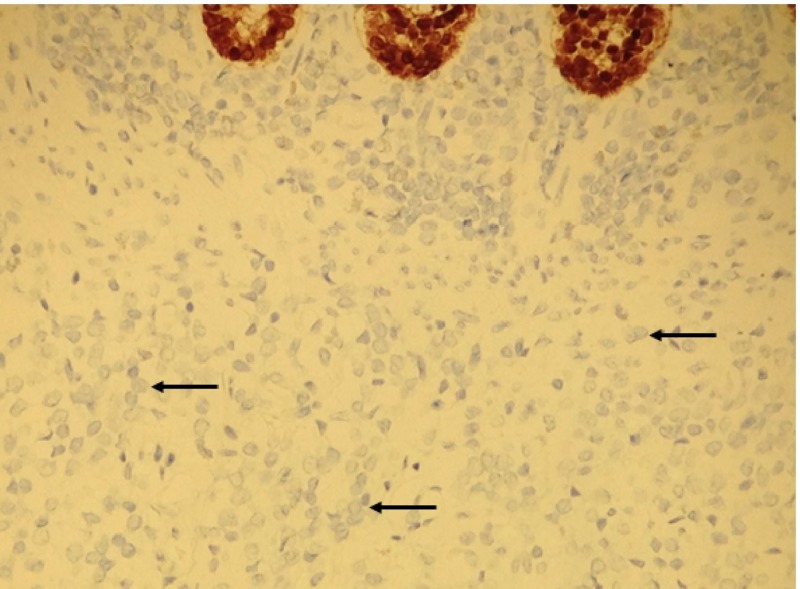
Immunohistochemistry fo CDX2 Tumor cells are negative with CDX-2 (arrows). Colonic crypt cells are positive (top of image).

**Figure 7:**
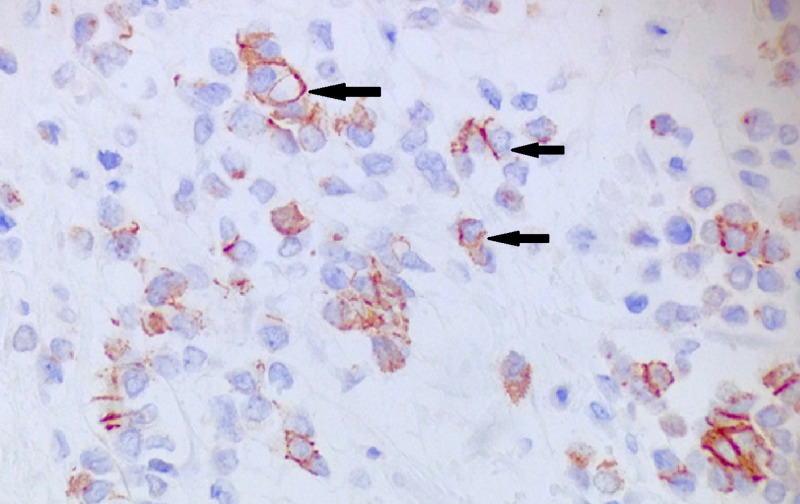
Immunohistochemistry stain for E-cadherin Positive membranous stain for E-cadherin (arrows).

The tumor was transmural with involvement of serosal fat through the lamina propria and percolated between the colonic crypts with six out of 11 lymph nodes positive. Immunohistochemical staining was positive for cytokeratin 7, estrogen receptor (Figure 3), and GATA-3 (Figure 4), and negative for cytokeratin 20 (Figure 5), caudal-related homeobox transcription factor - 2 (CDX-2) (Figure 6), MELAN-A, CD 56, and synaptophysin. This was consistent with metastatic breast cancer. Further stains were positive for E-cadherin (Figure 7), consistent with her history of invasive ductal carcinoma.

Given progression of metastatic invasive ductal carcinoma while on letrozole, she was transitioned to exemestane.

## Discussion

GI metastasis from breast cancer is rare [1-8]. Several case reports have examined the incidence and significance of this finding. Rajann et al. described a large clinical series of 2,500 patients with breast cancer and followed them for 18 years. They found only 17 patients, less than 1%, developed GI metastasis [1]. Nazareno et al. showed that the most frequent site of GI metastasis was the stomach [2] and that colon involvement by metastatic breast cancer is less common. Uygun et al. revealed that colon metastasis occurred in only 3% of cases (20 out of 720) [3].  

A few case reports have also demonstrated the rarity of metastasis of invasive ductal carcinoma to the colon. Although invasive ductal carcinoma is more common than invasive lobular carcinoma, the latter metastasize more frequently to the GI tract and retroperitoneum [2, 4-8]. Eljabu et al. reported that invasive lobular carcinoma accounts for 64% of GI metastasis. Even in mixed tumors, it is mostly lobular carcinoma that metastasizes to the GI tract [4]. Samo et al. discussed the tendency of lobular carcinoma to metastasize to the GI tract due to tropism of metastatic cells, while ductal carcinoma mostly spreads to the brain, liver, or lungs [5].

Based on the review of the literature, presentation of metastatic disease to the colon can be variable [1-3, 5]. It can be asymptomatic or it can present with signs of intestinal obstruction, such as nausea, vomiting, and abdominal distension [6]. Therefore, in patients with a history of breast cancer presenting with GI symptoms, metastatic disease must be considered in the differential diagnosis.

Metastatic disease can present synchronously, at the time of diagnosis of the primary cancer or metachronously, at a different time from the primary cancer diagnosis. Nazareno et al. specifically discussed metastatic disease from the breast primary to the colon occurring in as little as four months to as long as 30 years later [2]. Despite lengthy time intervals between primary diagnosis of breast cancer and the onset of subtle GI symptoms, clinicians should recognize the possibility that GI symptoms could be metastatic disease and that a comprehensive workup is required.

Although it is more common to have a second primary colorectal cancer than metastatic breast cancer to the colon, a biopsy is required to distinguish between the two diagnoses. Uygen et al. described that the resemblance to previous breast cancer and the absence of dysplasia in the adjacent colonic epithelium is suggestive of metastatic disease [3]. Nikkar-Esfahani et al. discussed that most metastatic breast cancers present as linitis plastica, a diffuse thickening of the colonic wall, and that the diagnosis can be missed if the biopsy was too superficial or the pathologist was not aware of the history of breast cancer [7]. Immunohistochemical stains are also used to distinguish these two different diagnoses. Cytokeratin biomarkers are useful in determining the origin of the tumor. Tumors that are negative cytokeratin (CK) 7 and positive CK20 suggest intestinal adenocarcinomas. GI cancers will usually also stain positive for homeobox protein CDX2. Comparatively, positive cytokeratin (CK) 7 and negative CK20 imply breast, lung, and ovarian adenocarcinomas, and metastatic breast cancer will also stain for CK 19, BRST-2, and GATA-3 [1, 8]. E-cadherin staining can determine whether the tumor is invasive ductal carcinoma or invasive lobular carcinoma of the breast. The loss of E-cadherin expression is a marker for a diagnosis of invasive lobular carcinoma, and E-cadherin positivity favors ductal differentiation [8-9]. Endocrine receptor status is also essential on the biopsied metastatic site as receptor conversion may change from the primary cancer. The accurate pathologic diagnosis of the disease allows for appropriate treatment plans. Systemic chemotherapy or hormonal therapy is preferred, and surgery should only be done for isolated metastasis or for palliation of symptoms [1, 4, 6-7].

While there have been advancements in treatment for metastatic breast cancer, colorectal metastasis usually indicates a poor prognosis [5, 7, 10]. Further, advanced age has a negative effect on survival. The median overall survival after diagnosis was 28 months, although survival up to nine years has been reported [10].

## Conclusions

In summary, this is a rare case report of metastatic invasive ductal carcinoma of the breast to the colon presenting as an obstructing mass. This case highlights that physicians must have exceptional clinical acumen when patients with previous malignancy present with a colorectal lesion. The differential diagnosis should always include metastatic disease until proven otherwise. Physicians should be aware of common metastatic patterns of malignancy; however, one must remember that disease sometimes presents in unique ways. The presentation and course of this patient's care will be a useful adjunct to the current literature for determining treatment and prognosis in similar cases.
